# Distinguishing Health Benefits of Eicosapentaenoic and Docosahexaenoic Acids

**DOI:** 10.3390/md10112535

**Published:** 2012-11-13

**Authors:** Fraser D. Russell, Corinna S. Bürgin-Maunder

**Affiliations:** Faculty of Science, Health, Education and Engineering, University of the Sunshine Coast, Maroochydore, Queensland 4556, Australia; Email: CBurgin@usc.edu.au

**Keywords:** LC *n*-3 PUFAs, omega-3 fatty acids, eicosapentaenoic acid, docosahexaenoic acid, resolvin D1, differential response, omega-3 fatty acid metabolites

## Abstract

Long chain omega-3 polyunsaturated fatty acids (LC *n*-3 PUFAs) are recommended for management of patients with wide-ranging chronic diseases, including coronary heart disease, rheumatoid arthritis, dementia, and depression. Increased consumption of eicosapentaenoic acid (EPA) and docosahexaenoic acid (DHA) is recommended by many health authorities to prevent (up to 0.5 g/day) or treat chronic disease (1.0 g/day for coronary heart disease; 1.2–4 g/day for elevated triglyceride levels). Recommendations for dietary intake of LC *n*-3 PUFAs are often provided for α-linolenic acid, and for the combination of EPA and DHA. However, many studies have also reported differential effects of EPA, DHA and their metabolites in the clinic and at the laboratory bench. The aim of this article is to review studies that have identified divergent responses to EPA and DHA, and to explore reasons for these differences. In particular, we review potential contributing factors such as differential membrane incorporation, modulation of gene expression, activation of signaling pathways and metabolite formation. We suggest that there may be future opportunity to refine recommendations for intake of individual LC *n*-3 PUFAs.

## 1. Recommendations for Daily Intake of *n*-3 PUFAs

Long chain omega-3 polyunsaturated fatty acids (LC *n*-3 PUFAs) are fatty acids with a long chain (20 carbons or more), with the first double bond located after the third carbon from the methyl end. LC *n*-3 PUFAs, including eicosapentaenoic acid (EPA) and docosahexaenoic acid (DHA), have long been proposed to bestow health benefits by improving blood pressure control, alleviating symptoms of rheumatoid arthritis and depression, as well as attenuating the progression of Alzheimer’s disease [1,2,3,4]. It is noteworthy that recent meta-analyses have reported no effect of LC *n*-3 PUFAs on incidence of fatal coronary heart disease and sudden death [5,6], in stark contrast to earlier trials that reported marked reductions in risk for these events [7,8]. Differences in trial design (open label versus randomized, double-blind, placebo controlled), baseline clinical management (for example, low *versus* high use of anti-thrombotic and anti-hypertensive medications), and absolute risk for adverse events (for example, patients with few versus many risk factors, including previous myocardial infarction, with diabetes and low use of statins), have been postulated to explain the divergent findings [9]. Dose is also an important determinant of patient outcome, where high doses only of LC *n*-3 PUFAs (for example, 2–4 g EPA + DHA/day) can provide cardiovascular benefit associated with serum triglyceride reduction [10]. Whilst evidence for the capacity of LC *n*-3 PUFAs to lower serum triglyceride concentration is particularly strong, improvements in patient outcome have additionally been ascribed to modulation of signaling pathways involved in inflammation and oxidative stress, improvement in endothelial function, and inhibition of platelet aggregation (see Section 2).

Although plant-derived α-linolenic acid (ALA) is obtained from dairy products and margarines [11] and can be enzymatically converted to EPA and DHA in humans, the process is inefficient (0.04%–2.84%), and restricted by high dietary intake of EPA, DHA and linoleic acid [12,13]. Whilst low delta-6 desaturase activity in humans contributes to poor conversion of ALA to EPA and DHA [14], levels of EPA and DHA can nonetheless be increased through dietary intake of LC *n*-3 PUFA-enriched foods or marine oil supplements containing these fatty acids [15]. Major sources of LC *n*-3 PUFAs in the diet of developed countries are fish, red meat and poultry [11,16,17], where combinations of these foods contribute high levels (>75% of the total intake derived from 29 different food groups) of DHA (fish and poultry), EPA (fish and red meat), and docosapentaenoic acid (DPA; red meat, poultry and fish) [11]. Many of the aforementioned benefits of LC *n*-3 PUFAs have been attributed to the intake of DHA and/or EPA.

Countries that have high dietary intake of fish, such as Japan, have populations that benefit from cardioprotective levels of LC *n*-3 PUFAs [18]. However, many populations consume amounts of LC *n*-3 PUFAs that are regarded as “adequate”, avoiding problems associated with deficiency, but not enough to provide health protection. Health authorities in these countries recommend increased intake of LC *n*-3 PUFAs to provide such benefits. For example, European health authorities recommend at least 0.45–0.50 g/day EPA + DHA to maintain good health [19,20,21]. The mean daily intake of EPA + DHA and ALA in Australian adults is 0.175 g and 1.07 g, respectively [22]. The Heart Foundation of Australia recommends a daily dietary intake of 0.5 g EPA + DHA plus 2.0 g ALA to lower the risk of coronary heart disease, 1.0 g EPA + DHA plus 2.0 g ALA for patients with documented coronary heart disease, and 1.2–4.0 g EPA + DHA for patients with elevated serum triglyceride levels [22]. Table 1 collates recommendations for dietary intake of LC *n*-3 PUFAs by selected National and International health and government organizations. Most of these organizations have issued recommendations for combined intake of EPA and DHA, but do not provide advice for the ratio of EPA to DHA. The French Agency for Food, Environmental and Occupational Health & Safety [20] is a notable exception, providing separate recommendations for dietary intake of EPA and DHA (Table 1). Following the American Psychiatric Association recommendation of 1.0 g/day DHA+EPA for the treatment of affective disorders ([23], Table 1), McNamara [24] proposed a 2:1 ratio of EPA:DHA for optimal patient management.

Differential associations for cell membrane EPA and DHA levels and resting state electroencephalographic (EEG) activity have been reported. DHA was associated with fast frequency EEG activity while EPA was associated with slow frequency EEG activity in 46 adolescent boys with attention deficit hyperactivity disorder [25]. Not surprisingly, the health benefits of the LC-*n*-3 PUFAs have often been ascribed to particular types of LC-*n*-3 PUFAs. For example, studies have reported blood pressure lowering effects of DHA but not EPA [26,27], while EPA has been reported to be more efficacious than DHA in reducing platelet activation [28,29]. The differential response to EPA and DHA suggests that an opportunity might exist to customize advice for the types or ratios of LC *n*-3 PUFAs to be used in the treatment of particular conditions or diseases. The implementation of such a strategy is certainly achievable, with a plethora of commercially available dietary marine oil supplements of defined LC *n*-3 PUFA composition [30]. For example, EPAX 5510 TG/N is an EPA-rich oil (EPA/DHA ratio of 5:1), while EPAX 1050 TG/N is a DHA-rich oil (EPA/DHA ratio of 1:5) [31]. Salunkhe *et al*. [32] recently identified a marine bacterial isolate that produces high concentrations of EPA (60% of total fatty acid content) at 30 °C, with no detectable production of DHA. The strategy may also be achievable by considering the dietary intake of fish and meat sources. For example, of fish high in LC *n*-3 PUFAs content, the ratio of EPA:DHA can range from ~1:2 for Atlantic Salmon and Ocean Trout to ~1:30 for Snook [33].

The purpose of this review is to examine studies that have reported heterogeneity with respect to effects of the LC *n*-3 PUFAs, chiefly EPA and DHA, and to examine some of the reasons for these differential responses. In particular, we review the impact of differential metabolism, membrane incorporation, modulation of gene expression, and activation of signaling pathways for EPA and DHA.

**Table 1:** Recommendations for Long chain omega-3 polyunsaturated fatty acids (LC *n*-3 PUFA) intake obtained from selected national and international health and government organizations.

Health Organization	Country	Recommendation	Ref.
National Heart Foundation	Australia	0.5 g/day EPA + DHA plus 2 g/day ALA to lower the risk of coronary heart disease; 1.0 g/day EPA + DHA plus 2 g/day ALA for patients with documented coronary heart disease; 1.2–4.0 g/day EPA + DHA for patients with elevated serum triglyceride levels.	[22]
American Heart Association	USA	≥2 fish meals/week plus oils rich in ALA in subjects without coronary heart disease; 1.0 g/day EPA + DHA for patients with documented coronary heart disease; 2.0–4.0 g/day EPA + DHA for patients with elevated serum triglyceride levels.	[34]
World Health Organization	International	0.2–0.5 g/day EPA+DHA to prevent coronary heart disease and ischemic stroke.	[35]
American Psychiatric Association	USA	1.0 g/day EPA + DHA for treatment of affective disorders.	[23]
1. National Health and Medical Research Council. 2. The Cancer Council Australia.	1. Australia & New Zealand.2. Australia	Adequate Intake for EPA + DHA + DPA: 0.09 g/day (women ≥ 19 years), 0.16 g/day (men ≥ 19 years). ALA: 0.8 g/day (women ≥ 19 years), 1.3 g/day (men ≥ 19 years). Intake of EPA + DHA + DPA to reduce risk of chronic disease: 0.43 g/day (women), 0.61 g/day (men).	[36,37]
Scientific Advisory Committee on Nutrition	UK	General nutrition, at least 0.45 g/day LC *n*-3 PUFAs.	[19]
French Agency for Food, Environmental and Occupational Health & Safety	France	General nutrition, 0.25 g/day EPA; 0.25 g/day DHA, 1% of energy intake ALA.	[20]
Health Council of the Netherlands	Netherlands	General nutrition, 0.45 g/day fish fatty acids.	[21]
International Society for the Study of Fatty Acids and Lipids	International	Cardiovascular health, ≥0.5 g/day EPA + DHA. General nutrition, 0.7% of energy intake ALA.	[38]

## 2. Evidence for Differential Responses to EPA and DHA

Although EPA and DHA are both long chain polyunsaturated fatty acids (EPA, C20:5n3; DHA, C22:6n3), the molecules are often reported to produce biochemical and physiological responses that are qualitatively and quantitatively different to each other. Table 2 provides a non-exhaustive overview of studies that report differential effects of EPA and DHA, and their metabolites. Each individual study represented in Table 2 was internally controlled, thus allowing direct comparisons of responses to the fatty acids under identical experimental conditions. This is an important point, since differences in experimental design between studies have sometimes led to inconsistent conclusions in regard to the effects of the LC *n*-3 PUFAs. When considering the blood pressure lowering effects of LC *n*-3 PUFAs for example, the reader needs to be cognisant of the effects of dose and duration of treatment, baseline characteristics of the subject for dietary intake of LC *n*-3 PUFAs and blood pressure, subject age, co-morbidities, and use of anti-hypertensive medications. It is nonetheless noteworthy that several studies in Table 2 reported response, and/or lack of response that was based on the examination of a single concentration of the LC *n*-3 PUFAs. In those studies it is possible that information obtained from construction of full dose-response curves to EPA and DHA might have shown the same qualitative response, albeit with different potencies and/or efficacies. For example, Yusufi *et al*. [39] reported an inhibitory effect of low concentration (10 μM) DHA, but not EPA on EGF-stimulated mitogenesis in mesangial cells. However, an inhibitory effect of EPA, quantitatively similar to that of DHA, was revealed when the LC *n*-3 PUFAs were used at a 10-fold higher concentration [39]. Similarly, studies that rely on single time-points for measurement of response may not capture changes that occur after short-term-, or more typically after long-term treatment regimens. This point is exemplified by the early incorporation (2–14 days) of EPA, but not DHA into erythrocyte membranes, with DHA incorporation occurring later (14–28 days) [40]. 

Interestingly, there are instances where EPA and DHA produce opposite responses, for example in the regulation of c-abl proto-oncogene, glutathione *S*-transferase A1, and breast cancer type 2 susceptibility protein gene expression [41], modulation of pulse pressure in a rat model of hypertension [27], and changes to resting heart rate in healthy human subjects [42] (Table 2). These examples suggest fundamental differences in the mechanism of action for the different LC *n*-3 PUFAs. Discussion of factors likely to contribute to the divergent responses to individual LC *n*-3 PUFAs is reported in Section 3.

**Table 2:** Summary of selected studies identifying differential effects of docosahexaenoic acid (DHA), eicosapentaenoic acid (EPA) and their metabolites.

Gene expression	Study	Summary of differences	Ref.
Genes regulating inflammation, the cell cycle, apoptosis	Jurkat T cells, 12.5 μM DHA (*n* = 3) or EPA (*n* = 3) for 1 day (compared to untreated cells).	CD27 ligand: DHA no change, EPA ↑. Fibronectin I: DHA ↑, EPA no change. Insulin receptor: DHA ↑, EPA no change. Microsomal Glutathione *S*-transferase I: DHA no change, EPA ↓. Cyclin-dependent kinase 4 inhibitor 2: DHA ↑, EPA no change. Phospholipase A2: DHA ↑, EPA no change. c-abl proto-oncogene: DHA ↑, EPA ↓. Glutathione *S*-transferase A1: DHA ↑, EPA ↓. Breast cancer type 2 susceptibility protein: DHA ↑, EPA ↓.	[41]
Cytokine mRNA expression	Lipopolysaccharide-stimulated human THP-1 macrophages. 100 μM DHA (*n* = 5–6) or EPA (*n* = 5–6) for 2 days.	Inhibition of TNFα, IL-1β, IL-6 mRNA: DHA > EPA. Cytoplasmic IκBα protein: DHA ↑, EPA no change.	[43]
UDP-glucuronosyl transferase IAI (UGTIAI) mRNA expression	Human hepatoma HepG2 cells, 50 μM DHA (*n* = 3) or EPA (*n* = 3) for 1 day (compared to vehicle). Some cells co-treated with 10 μM vitamin E.	UGTIAI mRNA: DHA no change (but ↓ with vitamin E), EPA ↓ (but ↑ to control levels with vitamin E).	[44]
Cannabinoid receptor 2 (CB2) and NAPE-PLD mRNA expression	MC3T3-E1 osteoblast-like cells. 10 μM DHA (*n* = 3–4) or EPA (*n* = 3–4) for 3 days (compared to vehicle).	CB2 mRNA: DHA no change, EPA ↓. NAPE-PLD mRNA: DHA no change, EPA ↓.	[45]
PPARγ and adiponectin mRNA	3T3-L1 adipocytes. 125 μM DHA (*n *= 3) or EPA (*n *= 3) for 1 day (compared to vehicle).	PPARγ mRNA: DHA ↑, EPA no change. Adiponectin mRNA: DHA ↑, EPA no change.	[46]
CYP2J2 mRNA expression	Human umbilical vein endothelial cells. 1, 10 μM DHA (*n *= 5) or EPA (*n *= 5) for 1 day (compared to vehicle).	CYP2J2 mRNA: DHA no change, EPA ↑.	[47]
Serum lipoproteins and LDL particle size	Overweight, non-smoking, mildly hyperlipidemic men. DB, RD, PC. 4 g/day DHA (*n *= 17) or EPA (*n *= 19) for 6 weeks.	HDL2-cholesterol: DHA ↑, EPA no change. HDL3-cholesterol: DHA no change, EPA ↓. LDL particle size: DHA ↑, EPA: no change.	[48]
Serum lipoproteins	Non-smoking, healthy men. DB, RD, PC. 3.6 g/day DHA (*n *= 72), 3.8 g/day EPA (*n *= 75) for 7 weeks.	HDL-cholesterol: DHA ↑, EPA no change. Apolipoprotein A–I: DHA no change, EPA ↓. Total cholesterol: DHA no change, EPA ↓. Accumulation into serum phospholipids: DHA > EPA. Δ6-Desaturation activity: DHA ↓, EPA no change. Δ5-Desaturation activity: DHA ↓, EPA ↑.	[49]
Serum lipoproteins	Non-smoking normolipidemic men and women. B, RD. 2.3 g/day DHA (*n *= 25) or 2.2 g/day EPA (*n *= 25) for 6 weeks.	HDL cholesterol: DHA ↑, EPA no change.	[50]
LDL particle size	Non-smoking hypertensive diabetic men and postmenopausal women. DB, RD, PC. 4 g/day DHA (*n *= 18) or EPA (*n *= 17) for 6 weeks.	LDL particle size: DHA ↑, EPA no change.	[51]
Triglyceride formation	Rat liver microsomes. 5–20 µM DHA-CoA (*n *= 4) or EPA-CoA (*n *= 4) for 10 min.	Triglyceride formation: DHA-CoA > EPA-CoA.	[52]
Lipid peroxidation	Rat C6 Glioblastoma cells. 100 μM DHA (*n *= 3) or EPA (*n *= 3) for 1–3 days.	Thiobarbituric acid production: DHA > EPA.	[53]
Endothelial cell migration	Cultured H5V endothelial cells. 100 μM DHA (*n *= 3) or EPA (*n *= 3) for 24 h.	Endothelial cell migration: DHA no change, EPA ↓.	[54]
Enzyme activity and membrane fluidity	Human cultured foreskin fibroblasts. 50 μM DHA (*n *= 6, membrane fluidity; *n *= 9 enzyme activity) or EPA (*n *= 6, membrane fluidity; *n *= 9 enzyme activity) for 4 days.	5′-nucleotidase activity: DHA ↑, EPA no change. Adenylate cyclase activity: DHA ↑, EPA no change. Fluorescence anisotropy: DHA ↑, EPA no change.	[55]
Mitogen signaling pathways	Jurkat T cells transfected with RasGRP. 10 μM DHA (*n *= 6) or EPA (*n *= 6) for 3 h.	Potentiation of PMA-stimulated ERK1/2 activity: DHA ↑, EPA no change *. * Result likely linked to differential incorporation of the LC *n*-3 PUFAs into diacylglycerol and not different affinities of the phospholipids for RasGRP.	[56]
Collagen-stimulated production of platelet thromboxane	Non-smoking men and postmenopausal women with type 2 diabetes mellitus. DB, RD, PC. 4 g/day DHA (*n *= 10) or EPA (*n *= 11) for 6 weeks.	Platelet thromboxane levels: DHA ↓, EPA no change.	[57]
Platelet aggregation	Healthy men and women. 1 μM DHA (*n *= 42) or EPA (*n *= 42) for 6 min. Healthy men; 1 μM DHA (*n *= 20) or EPA (*n *= 20) for 6 min (compared to ethanol control).	Aggregation to collagen; men and women: EPA ↓ > DHA ↓. Aggregation to collagen; men only: EPA ↓ > DHA ↓.	[29]
Platelet aggregation	Human platelets. 100 μM 4(*RS*)-4-F4t-NeuroP or 15-F3t-IsoP for 5 min prior to U46619.	Reversible aggregation to U46619: 4(*RS*)-4-F4t-NeuroP no change, 15-F3t-IsoP ↓. *Note: 4(*RS*)-4-F4t-NeuroP is a product derived from DHA*;* 15-F3t-IsoP is a product derived from EPA*.	[58]
Mean platelet volume and platelet count	Healthy men and women. RD, PC. 4 g/day DHA (*n *= 12) or EPA (*n *= 10) for 4 weeks.	Mean platelet volume: DHA no change, EPA ↓. Platelet count: DHA no change, EPA ↑.	[28]
Reactive oxygen species	Goat cultured neutrophils. 25–200 μM DHA (*n *= 6) or EPA (*n *= 6) for 0.5–2 h.	Cytochrome C activity in resting neutrophils: DHA ↓, EPA no change (0.5 h treatment). Cytochrome C activity in PMA-stimulated neutrophils: DHA ↓ > EPA ↓.	[59]
iNOS protein expression	Mouse RAW264 macrophages. 60 μM DHA (*n *= 3) or EPA (*n *= 3) for 1 day, then stimulated IFN-γ and LPS for 12 h (compared to untreated, stimulated cells).	iNOS/actin protein: DHA ↓, EPA no change.	[60]
Ca2+-induced opening of MPTP	Cardiac mitochondria from male Wistar rats. B. 2.5% caloric intake DHA (*n *= 8–9) or EPA (*n *= 8–9).	MPTP opening: DHA ↓, EPA no change.	[61]
Ischaemia-induced cardiac arrhythmias (SHR)	Spontaneously hypertensive rats. 0.5% w/w in the diet, up to 450 mg/kg/day; DHA (*n *= 10) or EPA (*n *= 10) for 5 weeks.	Ischaemia-induced cardiac arrhythmias: DHA ↓, EPA no change.	[62]
Blood pressure and thromboxane-like aortic constriction (SHR)	Spontaneously hypertensive rats during the development phase of hypertension. Blood pressure: 4.5% w/w in the diet; DHA (*n *= 8) or EPA (*n *= 8) for 12 weeks. Aortic constriction: 4.5% w/w in the diet; DHA (*n *= 5) or EPA (*n *= 5) for 12 weeks.	Blood pressure: DHA ↓ > EPA ↓. Aortic constriction: DHA ↓ > EPA ↓.	[62]
Salt-loading induced proteinuria (SHR)	Salt-loaded, stroke-prone spontaneously hypertensive rats with established hypertension. 4.5% w/w in the diet; DHA (*n *= 7) or EPA (*n *= 8) for 6, 9 and 12 weeks.	Proteinuria: DHA ↓, EPA no change.	[62]
Forearm blood flow (Human)	Overweight, non-smoking, mildly hyperlipidemic men. DB, RD, PC. 4 g/day DHA (*n *= 13) or EPA (*n *= 13) for 6 weeks.	Forearm blood flow: DHA ↑, EPA no change. Noradrenaline-mediated constriction of forearm microcirculation: DHA ↓, EPA no change.	[63]
Blood pressure and Heart rate (Human)	Overweight, non-smoking, mildly hyperlipidemic men. DB, RD, PC. 4 g/day DHA (*n *= 17) or EPA (*n *= 19) for 6 weeks.	Mean 24 h SBP: DHA ↓, EPA no change. Mean 24 h DBP: DHA ↓, EPA no change. Mean day SBP: DHA ↓, EPA no change. Mean day DBP: DHA ↓, EPA no change. Mean 24 h HR: DBP: DHA ↓, EPA no change. Mean day HR: DBP: DHA ↓, EPA no change. Mean night HR: DBP: DHA ↓, EPA no change.	[26]
Blood pressure and QT interval (male SHR)	Spontaneously hypertensive rats. 240 mg/day DHA (*n *= 6) or EPA (*n *= 6) for 8 weeks compared to normal fat diet.	Day SBP: DHA ↓, EPA no change. Night SBP: DHA ↓, EPA no change. Day pulse pressure: DHA ↓, EPA ↑. Night pulse pressure: DHA ↓, EPA ↑. Day QT interval: DHA ↓, EPA no change. Night QT interval: DHA ↓, EPA no change.	[27]
Heart rate	Non-smoking, healthy men. DB, RD, PC. 3.6 g/day DHA (*n *= 72) or 3.8 g/day EPA (*n *= 75) for 7 weeks (compared to control)	Resting HR: DHA ↓, EPA ↑.	[42]
Vascular tension (Rat)	Pre-contracted rat isolated aorta (untreated). 1 nM–100 μM 4(*RS*)-4-F4t-NeuroP (*n *= 4) or 15-F3t-IsoP (*n *= 4) dose-response curves.	Aortic contraction: 4(*RS*)-4-F4t-NeuroP no change, 15-F3t-IsoP ↑. *Note: 4(*RS*)-4-F4t-NeuroP is a product derived from DHA*;* 15-F3t-IsoP is a product derived from EPA*.	[58]
Antidepressant effect (Human)	Meta analysis, patients with depressive symptoms. DB, RD, PC. 28 studies.	Treatment of depression: EPA > DHA	[4]
Alzheimer disease (AD; Human)	815 subjects unaffected by AD; 65–94 years. Analysis of fish consumption using food frequency questionnaire; follow-up at 3.9 years.	Risk AD: DHA ↓, EPA no change.	[3]

↓, Effect of the fatty acid is to decrease; ↑, Effect of the fatty acid is to increase; B, Blinded; DB, Double-blind; RD, Randomized design; PC, Placebo controlled; SHR, spontaneously hypertensive rats; HDL, high density lipoprotein; LDL, low density lipoprotein; DHA, docosahexaenoic acid; EPA, eicosapentaenoic acid, PMA, phorbol 12-myristate 13-acetate, NAPE-PLD, *N*-acyl phosphatidylethanolamine-selective phospholipase D; MCP-1, monocyte chemotactic protein-1, MPTP, mitochondrial permeability transition pore.

## 3. Factors Contributing to Differential Responses to EPA and DHA

### 3.1. Regulation of Transcription Factors

LC *n*-3 PUFAs regulate gene transcription by binding to a variety of nuclear receptors, including the retinoid-activated nuclear receptor, RXRβ [64] (via association with the brain fatty acid-binding protein-7 transporter [65]), and peroxisome proliferator-activated receptor (PPAR) [66,67]. In 3T3-L1 adipocytes, 125 µM DHA upregulated PPARγ mRNA expression levels while 125 µM EPA was without effect [46]. PPARγ is a transcription factor for the adiponectin gene, and activation of PPARγ leads to increased synthesis of adiponectin. DHA stimulated secretion of greater amounts of adiponectin from adipocytes than EPA, and only the DHA response was blocked by an antagonist of PPARγ [46]. The authors concluded that there may be independent actions of EPA and DHA on PPARγ/adiponectin signaling in adipocytes [46]. The selectivity of DHA over EPA for PPARγ activation may not apply to all cell types, with evidence for stimulation of PPARγ activity by both EPA and DHA in human kidney-2 (HK-2) cells [68], and by EPA in human umbilical vein endothelial cells [47]. It is possible that differential effects of the LC *n*-3 PUFAs may be caused by metabolism of the LC *n*-3 PUFA to its metabolites. A number of oxidation products of DHA were found to be more potent activators of PPARγ than DHA [69,70], although metabolites resolvin D1 and resolvin E1 were without effect [71]. It is not yet known whether EPA and metabolites of EPA have differential activity at PPAR. The contribution of LC *n*-3 PUFA metabolites to differential responses observed for EPA and DHA are discussed in Section 3.4.

In addition to the direct effects of EPA and DHA on transcription factors, these LC *n*-3 PUFAs also indirectly modulate transcription factors. For example, LC *n*-3 PUFAs inhibit activity of the transcription factor, nuclear factor kappa B (NFκB) by attenuating phosphorylation and degradation of the inhibitory factor, IκB-α (reported for EPA [72]), and by inhibiting the recruitment of toll-like receptor 4 to lipid rafts (reported for DHA [73]). Although 100 µM DHA was more effective than 100 µM EPA in retaining IκB-α in the cytosol of lipopolysaccharide (LPS)-stimulated macrophages, EPA and DHA were equally effective at inhibiting LPS-stimulated NFκB/DNA binding activity [43].

### 3.2. Receptor-Mediated Effects of EPA and DHA

Free fatty acids were identified as endogenous ligands for the orphan G-protein coupled receptor, GPR120 following extensive ligand-binding screening [74]. GPR120 is expressed in gastrointestinal epithelial cells [74,75], and in macrophages [76], and its activation by LC *n*-3 PUFAs leads to secretion of glucagon-like peptide-1 from epithelial cells [74,77] and anti-inflammatory effects in macrophages [76]. Recent findings revealed short and long isoforms of GPR120, both of which were responsive to free fatty acids [78]. Although differential maximal efficacies for phosphorylation of these isoforms was reported in HEK293 cells exposed to α-linolenic acid and DHA, a comparison of EPA and DHA-mediated receptor phosphorylation was not made [78]. Nonetheless, it was speculated that the length and degree of saturation of the fatty acids may affect efficacy [78], raising the possibility that differences in efficacy might also be observed for EPA (20 carbons, 5 double bonds) and DHA (22 carbons, 6 double bonds). Whilst further studies are required to test this hypothesis, differences in amplitude of response to EPA and DHA have been reported previously. For example, serum response element-luciferase (SRE-luc) activity in HEK293 cells expressing GPR120 and the SRE-luc promoter appeared to be greater for cells treated with DHA than EPA [76]. Furthermore, secretion of glucagon-like peptide-1 from mouse colon was significantly elevated after intracolonic administration of DHA, but not EPA [77]. It is thus possible that the above interactions of EPA and DHA with GPR120 could in part explain the greater efficacy of DHA compared to EPA for the inhibition of release of pro-inflammatory cytokines from human macrophages [43] (Table 2). In that study, a single, high concentration of EPA and DHA was used (100 μM), and this was therefore likely to produce a maximal response (see [76]). Interestingly, potency does not appear to be different for EPA and DHA at GPR120. Potency of EPA and DHA for stimulation of calcium mobilization in HEK293 cells expressing GPR120, and promoter activity in HEK293 cells expressing GPR120 and SRE-luc promoter, were similar [74,76].

### 3.3. Incorporation of EPA and DHA into Phospholipids

Glycerophospholipids (phospholipids) are the main component of the cell membrane, and include structures such as phosphatidylcholine and phosphatidylserine. Dietary intake of EPA and DHA increases the LC *n*-3 PUFA content of phospholipids with an associated reduction in arachidonic acid levels [49,79,80]. The source of LC *n*-3 PUFAs may affect the extent to which EPA and DHA are incorporated into phospholipids. A recent study recruited healthy, young (20–50 years) men and compared incorporation of fish oil (EPA and DHA as re-esterified triglycerides or ethyl esters, with no free fatty acids) and krill oil-derived EPA and DHA (mainly bound in phospholipids, with 21%–22% free fatty acids) into plasma phospholipids [81]. A non-significant trend for higher incorporation of EPA and DHA from krill oil was reported [81]. Although the duration of the experiment was short (24 h), the investigators were careful to match the amount of EPA and DHA that was administered for the three preparations.

To investigate possible differential incorporation of EPA and DHA into membrane phospholipids, Judé *et al*. [79] fed dogs a diet containing a greater amount of DHA than EPA, for 8 weeks. As expected, the plasma concentration of DHA was greater than EPA. However, despite the intake of a DHA-rich diet, EPA was preferentially incorporated into erythrocyte and cardiac membrane phospholipids. The incorporation of EPA and DHA into phospholipids was also investigated in humans receiving dietary supplementation with LC *n*-3 PUFAs [14,82,83]. In these studies, EPA was also more efficiently incorporated into cholesteryl esters than DHA [14], and this was ascribed to a higher efficiency of lecithin-cholesterol acyltransferase (LCAT) activity for transfer of EPA from phosphatidylcholine to cholesteryl esters [82,84]. In contrast, DHA was preferentially incorporated into triglycerides [14], with DHA serving as a preferential substrate for diacylglycerol acyltransferase [52]. Interestingly, not all studies concur with this pattern of fatty acid metabolism. The supplementation of guinea-pigs with marine oils rich in EPA led to preferential incorporation of DHA into cardiac muscle total phospholipids [85]. Whilst species differences may be responsible for the different findings, this is unlikely to occur at the level of LCAT activity since LCAT activity in guinea-pig is intermediate between that reported in dog (lower activity than in guinea pig) and human (higher activity than in guinea pig) [86].

Both EPA and DHA decrease the liberation of arachidonic acid from phospholipids by inhibiting phospholipase A_2_ activity [87]. Arachidonic acid is a substrate for cyclooxygenase and lipoxygenase enzymes, and the competitive reduction in arachidonic acid by EPA and DHA inhibits the generation of the 2-series prostaglandins, and 4-series leukotrienes [88,89]. As described above, EPA is a better substrate than DHA for LCAT activity [82]. The displacement of arachidonic acid by EPA in phospholipids increases cyclooxygenase-2-mediated production of PGE_3_, and lipoxygenase-mediated production of LTB_5_, from EPA, with a concomitant reduction in levels of PGE_2_ and LTB_4_[90,91,92]. The PGE_2_-to-PGE_3_ and LTB_4_-to-LTB_5_ switching described by these investigators is likely to be of clinical importance in the *in vivo* modulation of disease since the EPA-derived products will tend to favour anti-inflammatory, anti-mitotic and anti-allergic activities [90,91,92].

Diacylglycerol is one of the lipid molecules into which EPA and DHA can be incorporated. This molecule has a key role in cell signaling, being a potent endogenous activator of conventional and novel subclasses of protein kinase C (PKC). Diets containing LC *n*-3 PUFAs lead to enrichment of these fatty acids in diacylglycerol [93], primarily in the *sn*-2 position [94]. Judé *et al*. [79] showed a preferential enrichment of cardiac membrane diacylglycerol with EPA compared to DHA in dogs that were fed a fish oil diet, while Madani *et al*. [56] showed preferential enrichment of Jurkat T cell diacylglycerol with DHA compared to EPA. In mice fed diets rich in EPA or DHA for 10 days, isolated splenic cells showed an apparent (data for *n* = 2 experiments only) differential enrichment of diacylglcerol with 18:1–22:6(*n*-3) and 18:1–20:1(*n*-9) (DHA fed mice) or 18:0–18:2(*n*-6) (EPA fed mice) [95]. The findings show that diets containing high levels of EPA or DHA can lead to differential incorporation of fatty acids, including LC *n*-3 PUFAs, into diacylglycerol. The question arises as to whether this type of response might translate to differential effects on PKC signaling. This was examined in an *in vitro* study that compared the effects of diacylglycerol containing EPA or DHA in the *sn*-2 position, on PKC activity [96]. Both EPA and DHA-containing forms of diacylglycerol stimulated concentration-dependent PKCα, βI, γ, δ and ε activity, however differential responses were not observed [96]. The implication of this finding is that whilst differential incorporation of EPA and DHA into membrane phospholipids may contribute to differences in health benefits of the LC *n*-3 PUFAs, this is unlikely to occur at the level of diacylglycerol/ PKC signaling.

### 3.4. LC *n*-3 PUFA Metabolites

**Figure 1:**
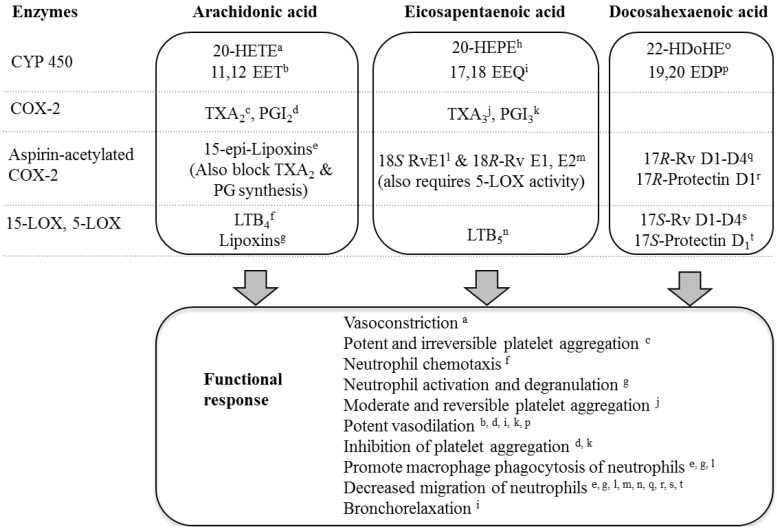
Metabolism of arachidonic acid, eicosapentaenoic acid and docosahexaenoic acid by cytochrome P450 (CYP450), cyclooxygenase-2 (COX-2), apirin-acetylated COX-2, and 15- and 5-lipoxygenases (LOX). Functional responses of metabolites are indicated [102,104,105,106,108,109,110,111,112,113,114,115,116,117,118,119,120,121,122,123]. 20-HETE, 20-hydroxyeicosatetraenoic acid; 11,12-EET, 11,12-epoxyeicosatrienoic acid; 20-HEPE, 20-hydroxyeicosapentaenoic acid; 17,18-EEQ, 17,18-epoxyeicosatetraenoic acid; 22-HDoHE, 22-hydroxydocosahexaenoic acid; 19,20-EDP, 19,20-epoxydocosapentaenoic acid; TXA_2_, thromboxane A_2_; TXA_3_, thromboxane A_3_; PGI_2_, prostacyclin; PGI_3_, prostaglandin I_3_; RvE1, resolvin E1; RvD1, resolvin D1; LTB_4_, leukotriene B_4_; LTB_5_, leukotriene B_5_.

LC *n*-3 PUFAs undergo enzyme-independent auto-oxidation under cell culture conditions, increasing with time and with the level of unsaturation of the fatty acid (DHA > EPA; [97]. Since oxidation products can have different activity to the parent LC *n*-3 PUFA, differential stability of EPA and DHA may impact on the pharmacological effect of these fatty acids. Multiple enzyme systems also convert LC *n*-3 PUFAs to metabolites that have biological activity [98,99] (Figure 1). The family of cytochrome P450 (CYP450) enzymes convert EPA to primary metabolites epoxyeicosatetraenoic acid (17,18-EEQ) and hydroxyeicosapentaenoic acid (20-HEPE), and DHA to epoxydocosapentaenoic acid (19,20-EDP) and hydroxydocosahexaenoic acid (22-HDoHE) [100]. Treatment of human subjects with 1.86 g EPA and 1.5 g DHA per day for 4 weeks caused a 4.7- and 2.1-fold increases in plasma levels of 17,18-EEQ and 19,20-EDP, respectively [101]. EPA is also converted to 18*S*- and 18*R*-resolvins E1 and E2 by aspirin-acetylated cyclooxygenase-2 (COX-2) and 5-lipoxygenase activity [102]. DHA is converted by aspirin-acetylated COX-2 to 17*R*-resolvins D1–D4 and 17*R*-protectin D1, and by 5- and 15-lipoxygenase to the 17*S*-series of these compounds [103,104,105].

Several studies have examined divergent activity EPA and DHA by studying the responses to their metabolites. Multiple isoforms of CYP450 contribute to the metabolism of EPA and DHA, and these can perform their catalytic function with different preference for the EPA and DHA substrates. For example, DHA is metabolized at a greater rate than EPA by CYP4F2, CYP4F3B, and CYP4F3A, while CYP4A11 and CYP2J2 preferentially metabolize EPA [99]. The response to the LC *n*-3 PUFAs will therefore be affected by expression levels of CYP450 isoforms in the tissues, as this will influence the degree to which EPA and DHA are metabolized. Importantly, the metabolites have different biological activities to each other, and to their parent LC *n*-3 PUFA (Figure 1). This point is exemplified by the markedly different potencies of EPA, DHA, and CYP450 metabolites of EPA and DHA for eliciting vasorelaxation. The potency of DHA for dilation of pre-constricted porcine coronary arterioles (apparent pEC_50_, 5.1) was ~336,000-fold lower than for its metabolite, 19,20-EDP (pEC_50_, 10.6) [106]. In contrast to the high potency of the DHA metabolite, the EPA metabolite 17,18-EEQ relaxed pre-constricted mouse mesenteric arteries with comparatively low potency (~pEC_50_, 7.0) [107].

Polymorphisms in the gene encoding for the CYP450 isoforms may contribute to variation in metabolism of EPA and DHA between individuals. Several polymorphisms have been identified in the CYP1A1 gene, including a CYP1A1.2 variant (Ile462Val) that has allelic frequency up to 10% in Caucasians, and up to 33% in Asians (see [124]). This variant form of CYP1A1 metabolizes EPA to 17,18-EEQ and 19-HEPE with a 2.1 and 5.2-fold higher efficiency than the wild type enzyme [124]. The ratio of efficiency of epoxygenation to hydroxylation is also different for the variant and wild-type enzymes [124]. Since the biological activity of the metabolites can differ (see above), polymorphisms may be expected to contribute to variability in differential effects of the LC *n*-3 PUFAs and their metabolites between individuals.

Differential responses have also been noted for the resolvins and lipoxins; families of autacoids with anti-inflammatory and pro-resolving activities. The resolvins mediate their effects, at least in part, through the activation of G protein coupled receptors; ALX/FPR2 and GPR32 for resolvin D1 and lipoxin A4 [71,108] and ChemR23 and BLT1 for resolvin E1 [108,125]. Some similarities exist in the functional response to the autacoids and their precursor molecules. For example, both resolvin D1 and DHA induced polarization of adipose tissue-infiltrating macrophages from an M1 to an M2 phenotype, as evidenced by the attenuation of M1 markers TNF-α and IL-6 and induction of the M2 marker Arg1 [126]. However, despite these similarities, differences between the LC *n*-3 PUFAs and their metabolites have also been noted. Whereas resolvin D1 potently induced macrophage phagocytic activity, DHA had the opposite effect, and whereas 1 nM resolvin D1 stimulated ROS production in peritoneal macrophages, DHA was without effect even when used at a 10,000-fold higher concentration [126]. It is thus possible that some of the differential responses reported for EPA and DHA might be attributable to the extent to which they are metabolized by the different enzyme systems to their endogenous autacoid metabolites. In addition to the differences observed between the metabolites and their parent LC *n*-3 PUFA, differences have also been reported for anti-inflammatory and pro-resolving activity between the various metabolites. For example, 17*R*-resolvin D1, formed by aspirin-acetylated COX-2, was more potent than lipoxygenase-generated 17*S*-resolvin D1 at inhibiting leukocyte infiltration in a mouse-model of peritonitis [105]. Differential kinetics for onset of anti-inflammatory and pro-resolving activity for the metabolites of arachidonic acid, EPA and DHA may contribute to some of the differential responses reported for these mediators. A stable analogue of 15-epi-lipoxin A4 inhibited pro-inflammatory cytokine production in a mouse-model of peritonitis, 4 h after administration [127]. At this time-point, resolvin E1 was without effect, however when assessed 12 h post-administration, it inhibited pro-inflammatory cytokine production with magnitude equal to, or greater than that observed for the lipoxin [127]. Few studies have examined the biological activities of the CYP450 metabolites, and further research into this area is warranted.

## 4. Conclusions

Long chain omega-3 polyunsaturated fatty acids (LC *n-*3 PUFAs), eicosapentaenoic acid (EPA) and docosahexaenoic acid (DHA), have been recommended for management of patients with wide-ranging chronic diseases, including coronary heart disease, rheumatoid arthritis, dementia, and depression. Although there is much evidence supporting uniformity of response to the individual LC *n-*3 PUFAs, there are also a plethora of studies that have shown qualitative and quantitative differences in response to EPA and DHA. In this review, we have explored some of the reasons for these differences. We propose that there are multiple factors that contribute to the differential effects of EPA and DHA, including differences in direct and indirect activation of transcription factors, impact of length, degree of saturation and stability of the fatty acid on efficacy, and differential efficiency for incorporation of the fatty acids into phospholipids. In addition, potency of the metabolites of EPA and DHA are often markedly different to the parent LC *n-*3 PUFA, and divergence in efficiency of enzymes to metabolize EPA and DHA can contribute to observed diversity in cellular response. We suggest that with improved understanding of the similarities and differences for EPA and DHA, an opportunity exists to customize recommendations for intake of EPA or DHA that can be tailored to the patient’s condition.
